# Construction of high-resolution recombination maps in Asian seabass

**DOI:** 10.1186/s12864-016-3462-z

**Published:** 2017-01-10

**Authors:** Le Wang, Bin Bai, Peng Liu, Shu Qing Huang, Zi Yi Wan, Elaine Chua, Baoqing Ye, Gen Hua Yue

**Affiliations:** 1 Molecular Population Genetics and Breeding Group, Temasek Life Sciences Laboratory, 1 Research Link, National University of Singapore, Singapore, 117604 Singapore; 2 Department of Biological Sciences, National University of Singapore, 14 Science Drive 4, Singapore, 117543 Singapore; 3 School of Biological Sciences, Nanyang Technological University, 60 Nanyang Drive, Singapore, 637551 Singapore

**Keywords:** Linkage map, Genome assembly, Recombination rates, GC content, Selective breeding

## Abstract

**Background:**

A high-density genetic map is essential for de novo genome assembly, fine mapping QTL for important complex traits, comparative genomic studies and understanding the mechanisms of genome evolution. Although a number of genomic resources are available in Asian seabass (*Lates calcarifer*), a high-density linkage map is still lacking. To facilitate QTL mapping for marker-assisted selection and genome assembly, and to understand the genome-wide recombination rates, we constructed high density linkage maps using three families and genotyping by sequencing.

**Results:**

A high-density consensus linkage map consisting of 8, 274 markers was constructed based on sex-averaged genetic maps. The genetic maps were then aligned and integrated with the current genome assembly of Asian seabass. More than 90% of the genome contig sequences were anchored onto the consensus genetic map. Evidence of assembly errors in the current genome assembly was identified. A fragment of up to 2.5 Mb belonging to LG14 was assembled into Chr15. The length of family-specific sex-averaged maps ranged from 1348.96 to 1624.65 cM. Female maps were slightly longer than male maps using common markers. Female-to-male ratios were highly variable both across chromosomes within each family and throughout three families for each chromosome. However, the distribution patterns of recombination along chromosomes were similar between sexes across the whole genome. The overall recombination rates were significantly correlated with genome-wide GC content and the correlations were revealed to be stronger in females than in males.

**Conclusions:**

These high-density genetic maps provide not only essential tools for facilitating de novo genome assembly and comparative genomic studies in teleosts, but also critical resources for fine mapping QTL and genome-wide association mapping for economically important traits in Asian seabass.

**Electronic supplementary material:**

The online version of this article (doi:10.1186/s12864-016-3462-z) contains supplementary material, which is available to authorized users.

## Background

A high-density genetic map is essential for facilitating genome assembly and examining the accuracy of de novo genome assembly [1]. De novo genome assembly achieved using massively parallel short read sequencing is not perfectly precise due to genome complexity resulting from ancestral vertebrate genome duplications (2R), gene duplications, and occurrences of transposable elements [2, 3]. Particularly, de novo genome assembly in teleosts is much more challenging, because of the additional fish-specific genome duplication event (FSGD or 3R) [4]. A high-density genetic map is particularly useful for determining the genetic basis of complex phenotypic traits, and studying the chromosomal structure variation and genome evolution by comparative genomic approaches [5]. Linkage maps can help identify genomic regions responsible for both economical and adaptive traits in evolutionary biology [6]. With the development of high-throughput SNP genotyping technologies [7, 8], high-density genetic maps become indispensable resources for investigation on genome-wide heterogeneity through the analysis of recombination rates because genomic regions showing higher levels of linkage are commonly exhibiting lower recombination rates [9, 10]. Although genetic maps with low marker density can also be useful in mapping QTL and aiding genome assembly, they are not sufficient to accurately detect the genetic loci and aid high-quality genome assembly. In particular, the architecture of genomic heterogeneity can only be detected at high resolution [11]. Thus, a robust high-density genetic map is considered as indispensable for studies in the genomic era.

Recombination is the most common genetic phenomenon and the basis for multiple biological contexts [12, 13]. It is considered to be associated with most genomic features, such as nucleotide diversity, GC content, gene expression regulations and epigenetic modifications, although the mechanism is still need to be elucidated [14]. Recombination interrupts linkage and thus allows more effective selection on multiple loci so as to generate complex phenotypic traits [9, 15]. However, elevated recombination would produce adverse effects from a perspective of animal breeding. Genomic regions with low recombination, e.g., holding together epistatically interacting alleles and inversions reducing recombination at specific areas, are also of great importance, as these regions are likely associated with selection [16]. Therefore, screening genome-wide recombination signatures and determining recombination hotspots and deserts are critically important issues for genomic studies. Besides in model species, investigations of recombination in non-model species are also needed in order to clarify both the general and species-specific patterns of recombination [17]. Due to the importance in genetic studies, high-density genetic maps have been constructed in a few economically important teleosts [18], e.g., Atlantic salmon [19–21], Coho salmon [22], channel catfish [23] and Japanese flounder [24], European sea bass [25], Nile tilapia [26], rainbow trout [27] and lake whitefish [28].

Asian seabass (*Lates calcarifer*) is one of the most important foodfish species in Southeast Asia and Australia [29]. The total production in these regions reached up to 75,000 t in 2012 [29]. This fish firstly matures as a male at 1–2 years of age and later reverses to a female at approximately 4 years old [29]. Sex reversal is a feature of species without specialized sex chromosomes [30]. Nevertheless, the genetic architecture underlying this interesting biological phenomenon remains to be elucidated. For the past decades, a lot of genomic resources have been developed in this species to facilitate genetic improvement, e.g., hundreds of thousands of genome-wide genetic markers derived from both coding and genomic sequences [31–33], high-density genetic linkage maps [34–36], a BAC-based physical map [37] and also a chromosomal-level genome assembly [33]. Several QTL responsible for growth-related traits [34, 38, 39] and viral disease resistance [36, 40] have also been identified and used for marker-assisted selection. Nevertheless, most of the linkage maps were constructed using microsatellites and they are of a resolution of less than 4000 markers. Thus, previous linkage maps are likely not enough to obtain confident examination of the accuracy of genome assembly and robust information on genomic heterogeneity of recombination [33, 35], and also to achieve accurate genomic prediction in the selective breeding programs of this species.

In this study, we constructed both family-specific and sex-specific genetic maps of Asian seabass, and generated a high-density consensus map by integration of these maps using high-throughput genotyping-by-sequencing (GBS) technology. These high-density genetic maps were firstly used to examine the accuracy of the de novo genome assembly of this species. In addition, the genome-wide recombination landscapes were investigated between sexes so as to understand the roles of genomic architecture in recombination, particularly for species without specialized sex chromosomes and with a sex reversal life history. Finally, the correlations between genome-wide recombination rates and nucleotide composition were examined to obtain the insights of the roles of genome composition on biological phenomenon. In total, these high density maps, provide essential tools for genomic studies and investigation of the genomic landscapes of teleost in correlation but not limited to sex reversal life history.

## Results

### Genotyping of genetic markers

After a series of filtering steps for removing samples with low sequence depth, 112, 118 and 118 progeny were retained for families Fam1, Fam2 and Fam3, respectively (Table 1). The average number of QC filtered reads for each progeny was more than 3.79 M. After excluding the markers showing significant segregation distortion, 2259, 3241 and 4025 SNPs were retained for genetic map construction for the three families, respectively. For microsatellites, 127 and 64 were used for linkage mapping for families Fam1 and Fam3, respectively, while for Fam2 no microsatellites were genotyped (Table 1). The number of common markers was relatively small with a proportion from 21.1 to 26.7% throughout families, which was significantly lower than that of the common catalogue loci between families, ranging from 52.9 to 79.0% (Table 1).

**Table 1:** Detailed information of three mapping families including the number of progeny, QC filtered reads, SNPs and microsatellites, common markers among families, catalogue loci and common catalogue loci for each family

Family	Progeny	QC filtered	No. SNPs	No. SSRs	No. common markers (%)	No. catalogue loci	No. common catalogue loci (%)
		reads (M)					
Fam1	112	4.28	2259	127	476 (21.1%)	121637	96054 (79.0%)
Fam2	118	6.15	3241	0	864 (26.7%)	153305	81083 (52.9%)
Fam3	118	3.79	4025	64	1018 (25.3%)	133199	77187 (57.9%)

### Construction of family- and sex-specific genetic maps

**Fig. 1:**
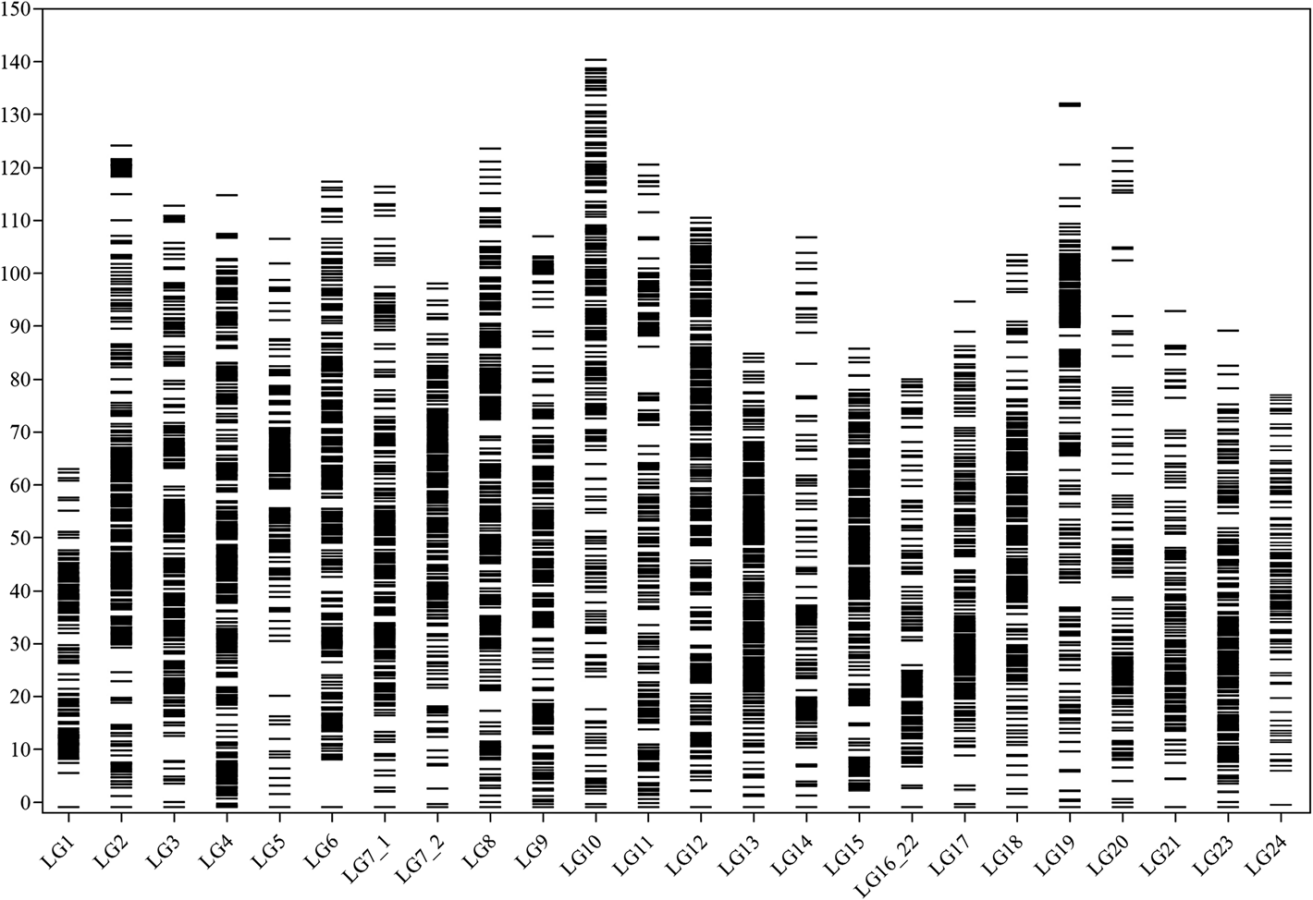
Distribution of genetic markers across the 24 linkage groups of the integrated consensus genetic map of Asian seabass

Three sex-averaged genetic maps were constructed using 2328, 3175 and 3836 markers for families Fam1, Fam2 and Fam3, with total lengths of 1348.96 cM, 1624.65 cM and 1412.91 cM, respectively (Table 2). The total number of markers with unique positions incorporated into the three family-specific maps was 2, 271 (97.6%), 3, 136 (98.8%) and 3, 686 (96.1%), respectively. The length of linkage group (LG), the number of markers with unique positions and the average marker interval based on the markers with unique positions for each individual LG showed significant variations throughout families. For example, LG1 of Fam1 was 68.67 cM in length with a marker interval of 0.82 cM, while LG1 of Fam2 and Fam3 were 31.40 cM and 23.03 cM in length, with marker intervals of 1.85 cM and 0.21 cM, respectively (Table 2). Detailed information for each sex-averaged genetic map is listed in Table 2. Using the common markers between families, a consensus genetic map consisting of 8, 274 markers was constructed by integration of the family-specific genetic maps, which included 7, 426 markers with unique positions (89.8%). The total length, average marker interval and resolution based on the markers with unique positions were 2546.86 cM, 0.37 cM and 4.52 cM/Mb, respectively. The number of markers in each LG ranged from 172 for LG24 to 499 for LG4 with an average of 345, while the marker interval was from 0.23 cM for both LG13 and LG15 to 0.71 cM for LG20 with an average of 0.31 cM, based on the markers with unique positions. In correlation to the physical map, the resolution of this consensus map ranged from 2.49 cM/Mb for LG1 to 7.65 cM/Mb for LG14, with an average of 4.52 cM/Mb (Table 3). The distribution of markers across LGs is shown in Fig. 1, where the largest marker interval is 11.07 cM in LG19. Estimated with the markers with unique positions, over 92.9% of the marker intervals were less than 1.0 cM, corresponding to 0.28 Mb of genome fragment.

**Table 2:** Summary statistics of sex-averaged genetic maps for three families of Asian seabass

Linkage	Fam1				Fam2				Fam3			
groups	Markers	Length	Intervals	cM/	Markers	Length	Intervals	cM/	Markers	Length	Intervals	cM/
	(unique)	(cM)	(cM)	Mb	(unique)	(cM)	(cM)	Mb	(unique)	(cM)	(cM)	Mb
LG1	84 (84)	68.67	0.82	2.67	17 (17)	31.40	1.85	1.22	121 (111)	23.03	0.21	0.90
LG2	110 (109)	54.75	0.50	1.80	197 (197)	50.39	0.26	1.66	228 (219)	69.91	0.32	2.30
LG3	191 (168)	76.31	0.45	3.25	191 (181)	57.13	0.32	2.43	151 (147)	47.18	0.32	2.01
LG4	121 (121)	52.98	0.44	2.07	169 (167)	50.65	0.30	1.98	282 (275)	69.06	0.25	2.70
LG5	224 (206)	73.38	0.36	2.53	120 (111)	28.04	0.25	0.97	89 (83)	42.81	0.52	1.48
LG6	114 (114)	37.86	0.33	1.36	201 (201)	85.86	0.43	3.07	62 (61)	108.22	1.77	3.87
LG7_1	135 (132)	48.86	0.37	2.10	77 (77)	75.14	0.98	3.23	178 (169)	56.29	0.33	2.42
LG7_2	51 (51)	54.37	1.07	3.91	184 (182)	66.93	0.37	4.81	208 (200)	60.82	0.30	4.37
LG8	118 (118)	60.90	0.52	2.35	164 (164)	85.50	0.52	3.30	188 (186)	43.09	0.23	1.66
LG9	103 (102)	44.25	0.43	1.92	153 (151)	61.98	0.41	2.70	187 (173)	90.31	0.52	3.93
LG10	125 (125)	71.35	0.57	2.55	112 (112)	110.48	0.99	3.95	101 (100)	62.65	0.63	2.24
LG11	49 (47)	63.48	1.35	2.73	114 (114)	72.04	0.63	3.09	216 (212)	47.47	0.22	2.04
LG12	108 (106)	71.91	0.68	2.58	182 (181)	82.07	0.45	2.95	279 (270)	68.53	0.25	2.46
LG13	82 (80)	42.15	0.53	1.55	187 (184)	72.60	0.39	2.66	220 (212)	91.68	0.43	3.36
LG14	58 (58)	50.60	0.87	3.60	96 (92)	106.58	1.16	7.57	70 (68)	22.42	0.33	1.59
LG15	86 (86)	49.89	0.58	1.62	196 (195)	49.79	0.26	1.62	174 (168)	70.08	0.42	2.28
LG16_22	62 (62)	48.95	0.79	1.71	107 (107)	55.12	0.52	1.92	134 (113)	38.07	0.34	1.33
LG17	48 (48)	47.32	0.99	1.83	45 (42)	69.35	1.65	2.68	209 (198)	59.07	0.30	2.29
LG18	91 (90)	39.71	0.44	1.44	133 (132)	82.26	0.62	2.97	218 (208)	74.23	0.36	2.68
LG19	37 (36)	54.01	1.50	2.81	155 (154)	42.52	0.28	2.22	26 (26)	31.27	1.20	1.63
LG20	184 (184)	67.33	0.37	2.74	124 (124)	82.01	0.66	3.34	106 (103)	48.05	0.47	1.96
LG21	34 (34)	56.80	1.67	2.39	60 (60)	74.72	1.25	3.15	74 (74)	75.58	1.02	3.18
LG23	64 (63)	49.66	0.79	2.73	87 (87)	59.81	0.69	3.29	223 (219)	64.54	0.29	3.55
LG24	49 (47)	63.48	1.35	3.20	104 (104)	72.27	0.69	3.65	92 (91)	48.58	0.53	2.45
Total	2328	1348.96	0.59	2.30	3175	1624.70	0.52	2.77	3836	1412.90	0.38	2.41
	(2271)				(3136)				(3686)			

Marker intervals were calculated based on the markers with unique positions within each linkage group

**Table 3:** Summary statistics of the integrated consensus genetic map of Asian seabass

Linkage groups	Physical length (Mb)	No. markers (unique)	Length (cM)	Intervals (cM)	cM/Mb
LG1	25.70	206 (182)	63.89	0.35	2.49
LG2	30.40	465 (440)	125.07	0.28	4.11
LG3	23.50	462 (375)	113.71	0.30	4.84
LG4	25.54	499 (464)	115.70	0.25	4.53
LG5	28.96	387 (276)	107.38	0.39	3.71
LG6	27.93	359 (348)	118.27	0.34	4.24
LG7_1	23.26	348 (319)	117.30	0.37	5.04
LG7_2	13.91	375 (339)	98.98	0.29	7.11
LG8	25.92	426 (404)	124.56	0.31	4.81
LG9	22.99	375 (318)	107.90	0.34	4.69
LG10	27.94	316 (307)	141.21	0.46	5.05
LG11	23.29	317 (284)	121.52	0.43	5.22
LG12	27.84	497 (443)	111.46	0.25	4.00
LG13	27.25	437 (375)	85.74	0.23	3.15
LG14	14.07	205 (182)	107.67	0.59	7.65
LG15	30.78	410 (371)	86.73	0.23	2.82
LG16_22	28.68	215 (175)	80.88	0.46	2.82
LG17	25.85	375 (334)	95.56	0.29	3.70
LG18	27.67	362 (323)	104.41	0.32	3.77
LG19	19.19	333 (306)	132.99	0.43	6.93
LG20	24.53	184 (175)	124.62	0.71	5.08
LG21	23.75	211 (199)	93.75	0.47	3.95
LG23	18.17	338 (323)	90.04	0.28	4.96
LG24	19.81	172 (164)	77.52	0.47	3.91
Total	586.95	8274 (7426)	2546.86	0.37	4.52

Marker intervals were calculated based on the markers with unique positions within each linkage group

A total of six sex-specific genetic maps were constructed for three families (Additional file 1: Table S1). The total number of markers mapped into these sex-specific maps ranged from 1373 for the Fam1 female map to 2637 for the Fam3 male map, while the total length was from 1246.96 cM for the Fam1 male map to 1422.83 cM for the Fam2 female map. The average marker interval based on markers of unique positions ranged from 0.52 cM for the Fam3 male map to 0.96 cM for the Fam1 female map. Two integrated sex-specific genetic maps were separately constructed based on the common markers of the independent sex-specific maps (Additional file 2: Table S2). The male and female integrated maps consisted of 5, 204 and 4, 771 markers, and had total lengths of 1955.90 cM and 2016.92 cM, respectively. Based on the markers with unique positions, the integrated male and female maps incorporated 4, 698 (90.3%) and 4372 (91.6%) markers, with an average interval of 0.42 cM and 0.46 cM, respectively.

### Integration of genetic maps with genome assembly

**Fig. 2:**
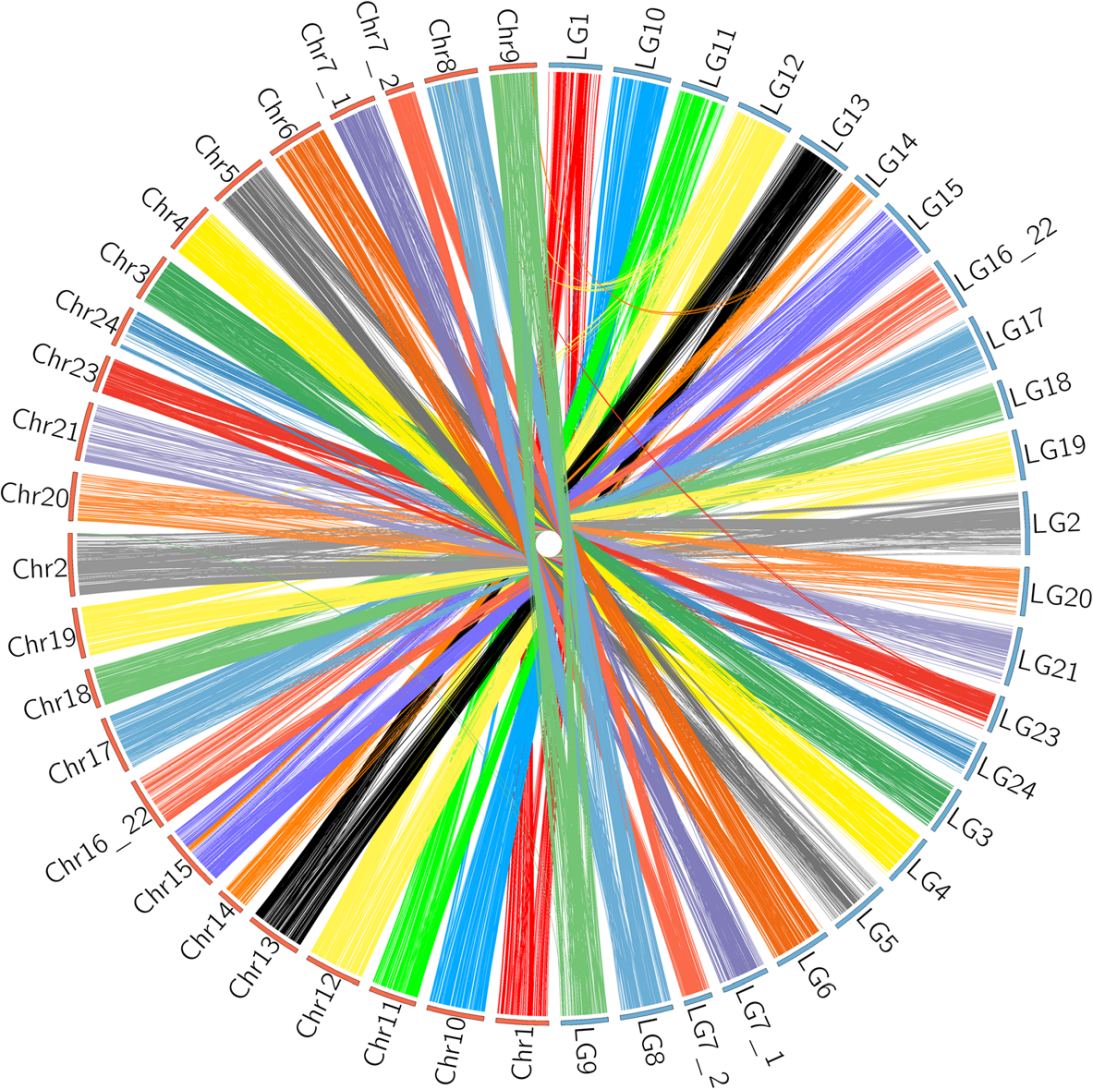
Genomic alignments between linkage groups and genome assembly of Asian seabass

Integration of the integrated consensus genetic map with the contigs assembly (genome assembly version 2) and scaffold assembly (genome assembly version 3) [33], revealed that 814 out of 3807 (21.4%) contigs and 259 out of 2964 (8.7%) scaffolds and/or contigs were anchored onto the map, accounting for 560.9 Mb (88.0% of 637.5 Mb, version 2) and 579.6 Mb (90.5% of 640.2 Mb, version 3) of the total length of reference genome, respectively. Integration of genetic map with genome assembly anchored additional 275 contigs with a total length of 19.9 Mb onto all 24 LGs. 7810 (94.4%) genetic loci were consistently identified in expected corresponding linkage groups and chromosomes, while 113 (1.4%) loci showed mismatches between specific LGs and the corresponding chromosomes of the reference genome (Fig. 2).

**Fig. 3:**
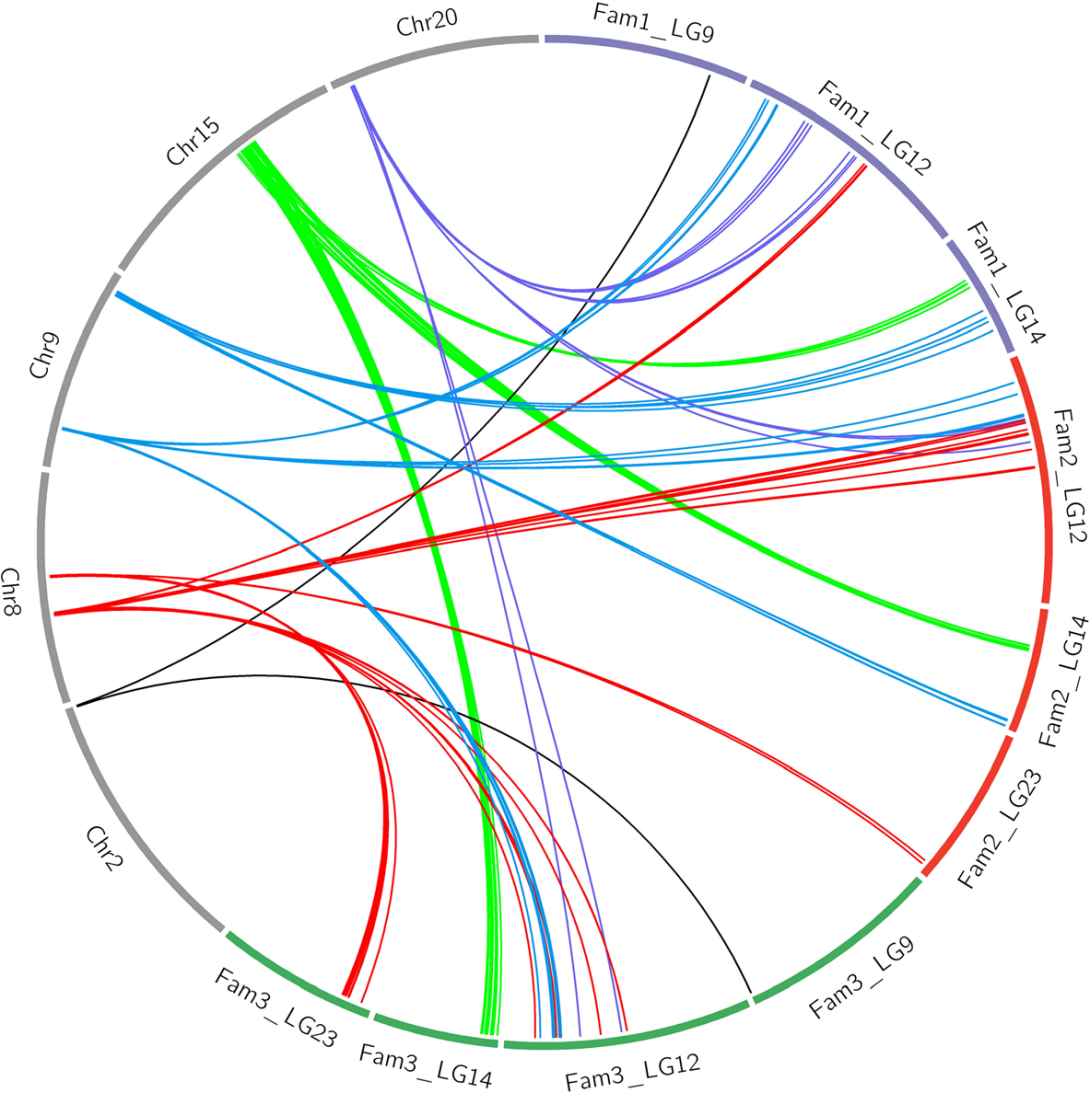
Mismatch between genome assembly and linkage groups of the three family-specific genetic maps

Genome synteny between chromosomes and LGs for each family-specific genetic map was further used to examine the mismatches. With the exception of the fragments that had no segregated markers for alignment between LGs and chromosomes, the mismatches were verified consistently by all three family-specific genetic maps, and distributed across five chromosomes of Asian seabass [33]: Chr2, Chr8, Chr9, Chr15 and Chr20 (Fig. 3). The largest fragment mismatch was observed for Chr15, where a genome fragment with a length of 2.5 Mb was anchored onto LG14. The most complicated genome syntenic relationships were observed for Chr8 and Chr9. Two fragments of Chr8 were mapped to LG12 and LG23, respectively, while two fragments of Chr9 corresponded to LG12 and LG14, respectively.

### Recombination heterogeneity between sexes

**Fig. 4:**
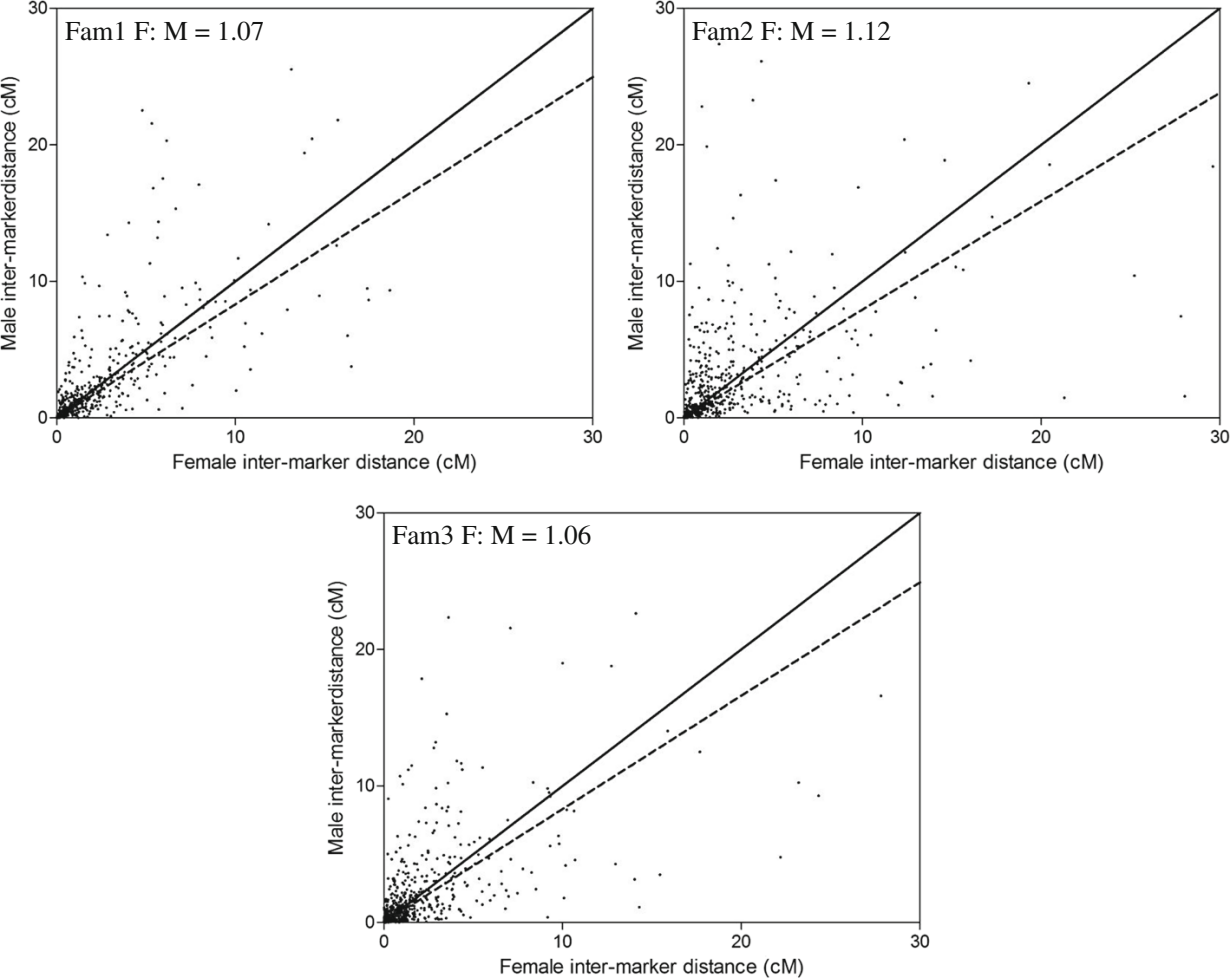
Distribution of the female against male inter-marker distances (cM) estimated based on common markers for three families of Asian seabass

**Fig. 5:**
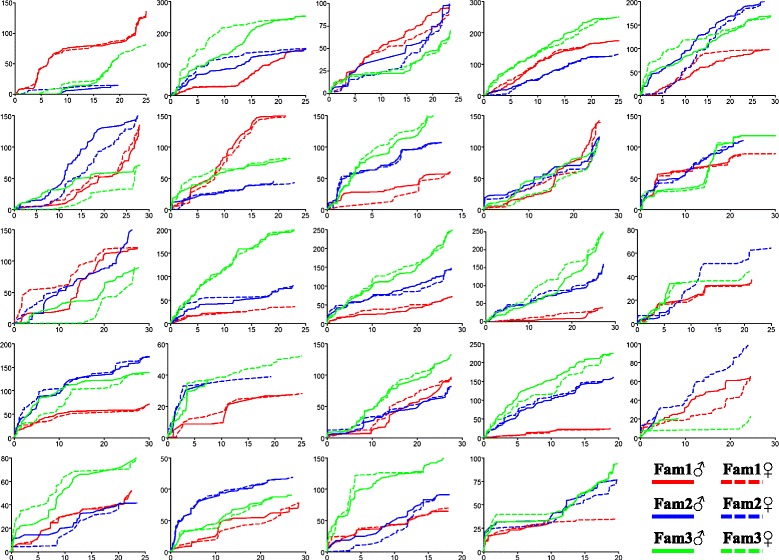
Accumulation of recombination rates (cM/Mb) along physical distance of each chromosome for sex-specific genetic maps of three families of Asian seabass

The average recombination rate throughout families at the whole genome level showed slight difference, ranging from 2.30 cM/Mb for Fam1 to 2.77 cM/Mb for Fam2 (Table 2). At the chromosome level, the overall recombination rate showed significant variations both across 24 linkage groups within each family (ranging from 1.36 to 3.91 cM/Mb, from 0.97 to 7.57 cM/Mb and from 0.90 to 4.37 cM/Mb for Fam1, Fam2 and Fam3, respectively) and throughout three families for each linkage group (e.g., ranging from 0.90 to 2.67 cM/Mb and from 1.36 to 3.87 cM/Mb for LG1 and LG6, respectively) (Table 2). For sex-specific recombination, we found the overall female-to-male ratios (F: M) throughout three families were not significantly deviated from 1, ranging from 0.94 for Fam3 to 1.09 for Fam2. The female genetic maps were slightly longer than the male ones for both Fam1 (F: 1303.98 cM vs M: 1246.96 cM) and Fam2 (F: 1422.83 cM vs M: 1308.23 cM), whereas the male map was slightly longer than the female map for Fam3 (M: 1332.97 cM vs F: 1249.26 cM). For every linkage group, we observed significant differences in female-to-male (F: M) ratios throughout families and also significant differences in the number of markers between sexes within each family, e.g., LG11 in Fam1 and LG19 in Fam2 (Additional file 1: Table S1). However, due to the markedly different number and sets of genetic markers used for individual map construction, we could not directly compare the recombination rates between sexes. For this reason, all pairs of the common markers shared between sexes for every family were used to estimate and compare the recombination ratios. We observed the overall recombination rates were slightly higher in females than in males throughout all three families (overall F: M, 1.08; *P* < 0.001, paired *t*-test; Fig. 4). However, using common markers, we found 18 out of 24 linkage groups did not consistently deviate from F: M = 1 throughout the three families (Additional file 3: Figure S1). The distribution patterns of recombination along chromosomes between sexes were in good agreement for most of the chromosomes within each family (Fig. 5). Although significant differences in the distribution patterns of recombination can be detected in some LGs, e.g., LG2, LG5, LG6, LG10 and LG19 (*P* < 0.001; Fig. 5), such differences are most likely caused by the variations of the number of markers within these linkage groups (Additional file 1: Table S1).

### Correlations between recombination and GC content

In comparison to sex-averaged genotype segregation that includes influences from both sexes, genomic sequence features are more directly associated with sex-specific segregation events. Therefore, correlations between genome-wide recombination rates and GC content were firstly evaluated using sex-specific genetic maps. We observed a significantly positive association between recombination rates and GC content for each sex-specific genetic map throughout all families (Table 4). Interestingly, we detected that the correlations were significantly stronger in females than in males (*P* = 0.02, Chi-squared test). The locus intervals with GC content of < 40% showed higher recombination rates in males than in females, while locus intervals showing GC content of > 40% had higher recombination rates in females than in males (Additional file 4: Figure S2). However, we did not find any difference in GC content between the chromosomes that showed consistent F: M ratios (>1 or < 1), e.g., LG5, LG6, LG15, LG16_22, LG18 and LG24, and the other chromosomes throughout the three families (Additional file 3: Figure S1).

**Table 4:** Correlation tests between GC content and recombination rates for each sex-specific linkage map in Asian seabass, examined using both Pearson and Spearman correlation tests

Linkage	Pearson test		Spearman test	
map	R	*P* value	R	*P* value
Fam1 ♀	0.377	< 0.001	0.337	< 0.001
Fam1 ♂	0.327	< 0.001	0.251	< 0.001
Fam2 ♀	0.332	< 0.001	0.598	< 0.001
Fam2 ♂	0.324	< 0.001	0.497	< 0.001
Fam3 ♀	0.434	< 0.001	0.328	< 0.001
Fam3 ♂	0.357	< 0.001	0.237	< 0.001

## Discussion

### Genetic maps

In this work, genetic maps were computed for multiple families using both GBS based SNPs and microsatellites. A consensus genetic map of the highest resolution to date in Asian seabass was constructed. This strategy for construction of high-density integrated genetic map has been applied in some fish species, e.g., common carp [41] and channel catfish [23]. The genetic map will be useful for a wide range of genomic studies.

With the availability of different types of high-throughput SNP genotyping platforms, construction of a reliable high-density genetic map is of great importance for downstream studies, such as genome-wide association mapping for complex traits and accurate comparative genomics [7, 35]. Nevertheless, issues from missing genotypes and genotyping errors are still considerations for all the current available SNP genotyping approaches, as they could lead to incorrect ordering of genetic markers in linkage mapping and also inaccurate estimation of recombination in genetic maps [42]. Compared to the other platforms, e.g., SNP arrays, GBS particularly suffers more from the above disadvantages [7, 43]. Thus, it is critical to obtain accurate and effective data sets in this study to construct confident high-density genetic maps.

As revealed in our previous studies using GBS, construction of genetic maps was achievable [32, 35]. Here, we adapted not only much stricter filtering criteria for SNP genotyping, but also more data control processes so as to generate high confidence genetic maps. Firstly, reference based sequence alignment and higher sequence coverage were applied to increase the accuracy of SNP recovery [44]. We only retained the samples of high genotyping quality in each family. Moreover, loci with any evidence of Mendelian distortion, low call rates and being from paralogs that tend to cause errors were removed [45]. In addition, we applied a strategy of using multiple families for cross validation of the mapping results. As revealed by our data, 8274 (94.6%) among all the 8746 loci were successfully mapped into the integrated consensus genetic map, suggesting high confidence of the results of linkage mapping throughout all families. Last but not least, microsatellites with relatively few missing genotypes (< 5%) were also used for linkage mapping. Throughout all constructed genetic maps, these loci showed little deviation between relative genetic positions and physical positions, also indicating that linkage mapping based on GBS in this study was robust. Nevertheless, it was also evident that the number of common markers shared among families was relatively smaller in GBS studies, compared to both microsatellites [34] and SNP chip based studies [23, 46]. The low number of common markers was caused by both the non-informative markers between families and the RAD loci none identified in all families. The proportion of common markers, to some extent, could be a challenge for integrating genetic maps, although the influence remains to be elucidated.

The high-density consensus genetic map had a total length of 2546.86 cM, showing non-significant difference with the multi-family based genetic map (2411.5 cM) of Asian seabass. As the densest map ever constructed for Asian seabass, it incorporated 8274 markers with an average marker interval of 0.31 cM and covered more than 90.5% of the reference genome. More than 92.9% of pairwise locus distances of unique positions within this map were < 1.0 cM (~ 0.28 Mb). In comparison, the genetic map of the highest resolution (3321 SNPs and 0.52 cM) constructed in previous studies covered 84.6% of the reference genome, with 83.8% of pairwise locus distances within 1.0 cM (~ 0.41 Mb) [35]. Thus, the current genetic map is a substantial improvement to previously published maps in Asian seabass [34, 35] and provides an excellent resource for fine mapping QTL in Asian seabass. For individual family-specific maps, the total length varied from 1348.96 cM to 1624.65 cM, which also showed no difference from a previous map based on GBS with a total length of 1577.67 cM [35]. Similarly, there was also no difference for the length of sex-specific maps between this study and previous studies [34, 35]. All these data suggest robustness of the genetic maps in this study. However, the order of markers in the genetic maps should be treated with caution because the meiosis or the population size in this study might be not enough to accurately order all the available markers. Therefore, the ordering of contigs from the reference genome should be examined in future studies. In QTL studies, a high density genetic map is indispensable. Nevertheless, it also should be noted that the number of crossovers or individuals used for mapping is another critical issue influencing the QTL mapping resolution, which is particularly important in mapping complex traits [47].

### Applications of the recombination map in genome assembly

All SNPs used for mapping were generated by alignment against the reference genome [33], thus the high-density genetic maps showed particular importance in facilitating and verifying the assembly of the whole genome and in comparative genomic studies of Asian seabass [1]. The genome assembly was integrated with the consensus linkage map using both SNP and microsatellite loci information. More than 90% of contig sequences (579.6 Mb) were mapped onto the genetic map, which was slightly higher than that in the aquaculture species, European seabass (575 Mb, 86%) [48] and channel catfish (867.4 Mb, ~ 90%) [23]. The integration assigned additional 275 contigs into 24 LGs. Although we observed a rather high level of consistency or synteny between chromosome assembly and the genetic map, 1.4% of loci in the genetic map were identified matching to five non-corresponding chromosomes. These mismatches were further verified and were confirmed by the consistent results of integrations between chromosome assembly and each of the three family-specific maps. All these data possibly suggest errors of assembly at least for five chromosomes (Chr2, Chr8, Chr9, Chr15 and Chr20) in the current genome assembly of Asian seabass [33]. Interestingly, all genetic maps in this study consistently suggest that a fragment of up to 2.5 Mb that was possibly from Chr14 was mistakenly assembled into Chr15. All these possible assembly errors should be noted in the future fine-scale mapping of QTL and high-resolution comparative genomic studies. Actually, it is almost impossible to avoid assembly errors in genome sequencing projects, particularly using massively parallel short read sequencing, due to the complexity of genomes [49]. In teleosts, the genome has experienced duplication two to three times [4, 50]. The occurrences of repetitive fragments, resulting from retention of duplicate genes and transposons, significantly increase the difficulties of assembling an accurate genome [3]. The accuracy of de novo genome assembly is still a major issue that needs to be improved in the genomic era [49]. Here, the integration of genetic map with reference genome provides excellent resources for comprehensive comparative genomic analyses, fine mapping QTL, and even positional cloning of candidate genes.

### Patterns of sex-specific recombination

Overall, the sex-specific recombination rates were revealed to be highly variable both across 24 chromosomes within each family and throughout three families for each chromosome, although the female maps presented slightly higher recombination rates than the male maps as revealed using common markers, in Asian seabass, a species without heteromorphic sex chromosomes [29]. The average recombination rates ranged from 2.4 cM/Mb to 2.8 cM/Mb throughout three families, comparable to some representative aquaculture species: Atlantic salmon (~ 2.6 cM/Mb) [19], channel catfish (~ 3.5 cM/Mb) [23] and bighead carp (~ 1.8 cM/Mb) [51]. Slight differences in recombination rates within species can be partially explained by the genome sequence variations (e.g., structural and copy number variations) and composition heterogeneity (e.g., GC content) [14, 17, 20].

Sex-specific recombination is a common biological phenomenon and is widely studied in different biology systems [52–55]. For species with chromosomal mechanisms of sex determination, the homogametic sex typically has relatively higher level of recombination than the heterogametic sex [14, 23, 53, 54]. Although sex-specific recombination is also common in species without chromosomal mechanisms of sex determination [17, 19, 56, 57], the comprehensive patterns for specific species are so far still not clear. Here, Asian seabass, lacking specialised heteromorphic sex chromosomes, was also observed having a differential pattern of sex-specific recombination. The overall pattern also showed a slightly female biased recombination (F: M, 1.08), which was similar to species both with specialised heteromorphic sex chromosomes, such as channel catfish [23], chicken [58], pig [14] and mouse [59] (F: M, ~ 1.26–1.73), and without heteromorphic sex chromosomes, such as Atlantic salmon [19, 20], grouper [60], zebrafish [57] and oyster [17] (F: M, ~ 1.03–2.74). This result was consistent with our previous study in Asian seabass using GBS [35], but different from another study using microsatellites [34], which showed a slightly male biased recombination. As revealed in this study, the overall female- and male-specific recombination rates were only slightly different. Thus, differences in marker density for specific LGs are likely to have significant influence on the overall recombination rates when using low-density genetic maps [57]. Besides, different markers used in comparisons can also lead to biased results, particularly in species with slightly different sex-specific recombination rates [59]. For example, Fam3 showed slightly higher recombination in males using the whole data set. However, when using only common markers, we observed a significant female biased recombination (F: M, 1.06). Here, our data also suggest that adequate marker coverage and common markers between sexes are indispensable to evaluating sex-specific recombination in future studies [59].

Study on the sex-specific recombination across chromosomes for species without differentiated sex chromosomes and with a sex reversal life history is of particular interest for understanding the mechanisms for biological features associated with meiosis and sex determination systems of these species [17, 61]. Here, we found the distribution patterns of recombination along chromosomes between sexes were in high agreement for most of the chromosomes within each family, e.g., LG1, LG4 and LG7_1. Such sex-specific distribution pattern of recombination was similar to that found in a previous study in pig [14], but extensively different from that in oyster, a species also without differentiated sex chromosomes [17]. Nevertheless, the chromosomes both showing good and none agreement in the distribution patterns of recombination along chromosomes between sexes, did not consistently and significantly deviate from F: M = 1 throughout three families. This was different from some species with specialised heteromorphic sex chromosomes, e.g., pig [14]. Thus, the level and the distribution patterns of recombination might be highly diverse among organisms [14, 17, 23]. It likely suggests that sex-specific recombination is associated with both universal and species-specific mechanisms, which need to be studied using ultra-resolution genetic maps.

Sex-specific recombination is suggested to be associated with genome-wide signature of GC content [61, 62]. Consistent with the other studies in mammals, birds, invertebrates, yeast and plants [9, 63–65], we also observed a significant correlation between GC content and recombination rates for both sexes. Interestingly, the correlation was significantly stronger in females than in males, which was consistent with the pattern that females have a slightly higher overall combination rates than males. Taking into consideration that the studied organism is a species with the feature of sex reversal, these results mean that the recombination rates would change across the genome for one individual at different life history stages. Thus, it is much likely that the pattern of recombination is not determined directly by genomic factors, but is due to the imprints resulting from sex determination and differentiation as discussed above [62, 66]. Studies on genome-wide recombination still have great importance in comparative genomics for various species [67].

## Conclusions

We constructed a high-density genetic map with 8274 markers using multiple families and a GBS approach. Some misassembles in the current genome assembly of Asian seabass were identified using high-density linkage maps. The recombination rates were highly variable both across chromosomes within each family and for each chromosome throughout families. The overall recombination rates were slightly higher in females than in males. Most of the chromosomes showed a good agreement in the distribution pattern of recombination along chromosomes between sexes. In addition, the recombination rates were significantly correlated to the genome-wide GC content for both sexes with an evidence of female bias. These data provide critical genomic resources for genome assembly, mapping QTL for economically important traits to accelerate genetic improvement in Asian seabass and comparative genomic analysis.

## Methods

### Mapping populations and genotyping-by-sequencing

Three full-sib families were used for construction of genetic maps. In detail, Fam1 was a backcross generated by a F_2_ male offspring crossing its F_1_ female parent [40], while both Fam2 and Fam3 were independent F_2_ populations from two different parents set up as described in our previous study [35]. For each family, two parents and 192 progeny randomly selected and genotyped with microsatellites and genotyping-by-sequencing using the ddRADseq approach [68].

Genomic DNA was isolated from fin tissue using the salt precipitation method [69]. 149 microsatellites, almost evenly covering the genome, were genotyped according to a previous study [40]. GBS libraries were constructed according to our previous method [35]. In brief, 300 ng genomic DNA was digested with *Pst*I-HF and *Msp*I restriction enzymes (New England Biolabs, USA) and was then ligated with adaptors using T4 ligase (New England Biolabs, USA). The ligation products were pooled for size selection of 300–500 bp by running gels, after clean up with QIAquick PCR Purification Kit (Qiagen, Germany). The libraries were enriched using PCR with Phusion® High-Fidelity DNA Polymerase (New England Biolabs, USA). After a final clean up using QIAquick PCR Purification Kit (Qiagen, Germany), ddRADSeq libraries were sequenced using a NextSeq 500 platform (Illumina, USA) for either paired-end (2 × 150 bp) or single-end (1 × 150 bp).

Raw reads processing and SNP genotyping were conducted using the software package Stacks v1.34 [70]. Reads were trimmed to 120 bp and those with any uncalled base were removed. QC filtered reads were aligned against the reference genome of Asian seabass [33] using the program BWA with a maximum of two mismatches [71]. Alignments with multiple genome targets were excluded from further analysis. Reference aligned reads were used for stacks assembly using pstacks implemented in the package Stacks v1.34 [70]. The stacks assembled for the parents of the three families were used to construct a catalogue with the program cstacks Stacks v1.34 [44]. The catalogue of loci was then used as reference for SNP discovery and genotyping for each family with the program populations implemented in Stacks v1.34 [70]. SNP filtrations were conducted similarly to our previous study with some modifications [35]. In brief, RAD tags with any SNP of > 2 alleles and showing heterozygosity of > 0.5 were removed [72]. A minimum of 10 × sequence depth and a genotyping success of > 85% of the individuals in each family were used for SNP genotyping. In order to reduce the number of missing genotypes, samples with low sequencing depth were ruled out for each family. Only one SNP per tag was retained for map construction.

### Genetic map construction

All genotypes were used for goodness-of-fit tests for Mendelian segregation distortion using *χ*2-analysis. Loci that showed any signal of segregation distortion at the significance level of 0.05 were removed. The program JoinMap 4.1 [45] was used for map construction, where an LOD of 10 was used for grouping of nodes. The marker distances in each linkage group were determined using the regression mapping algorithm and the Kosambi mapping function. The pseudotestcross strategy was used for construction of sex-specific genetic maps [73]. It assumes that markers with 1:1 ratio segregation imply one parent is heterozygous while the other is homozygous, thus the male and female genotypes can be obtained. Markers which were heterozygous in the male parent but homozygous in the female parent were used to construct the male genetic map, while markers which were heterozygous in the female parent but homozygous in the male parent were used to construct the female genetic map. Consensus genetic maps were constructed by integrating individual genetic maps according to the genetic positions of the common markers among individual maps using the program MergeMap [74].

### Integration of genetic maps with genome assembly

All mapped microsatellites with flanking sequences were aligned against the genome assembly, including all contigs and scaffolds, of Asian seabass [33] using BLAST with a cutoff of 1E^−10^ and a minimal sequence identity of 95%. The syntenic relationships between genetic maps and the genome assembly were constructed and the integration was achieved by anchoring the contigs and scaffolds onto the genetic maps according to marker positions. The program Circos [75] was used to visualize the genomic synteny and integration between genetic maps and genome assembly. The accuracy of genome assembly was examined by integration of both consensus and individual genetic maps with the genome assembly.

### Analysis of recombination heterogeneity

In order to investigate recombination heterogeneity across chromosomes between sexes, recombination rates were estimated for both sex-specific and family-specific genetic maps. Recombination fractions for locus intervals between marker pairs were calculated using the program JoinMap 4.1 [45] and were further mapped along the physical map in cM/Mb. Sex-specific recombination heterogeneity throughout independent chromosomes was estimated based on the locus intervals of common markers between sexes for each individual family. The significance was examined using the goodness of fit tests according to Ott’s method [76], followed by Bonferroni corrections for multiple comparisons. Due to limited number of common markers, recombination heterogeneity among families was examined by comparing the trends of variations for recombination rates along physical distance. For visual comparisons between sexes and among families, recombination rates for continuous locus intervals were plotted against physical distance along each individual chromosome.

### Analysis of genomic features of recombination

The possible correlations between average recombination rates and genome-wide nucleotide compositions, i.e., GC content, were estimated. Calculation of GC content was based on the full reference genome sequences within locus intervals. The correlations were assessed using both the Pearson and Spearman correlation tests. Compared to family-specific maps that involve segregations from both sexes, sex-specific maps are more directly associated with genome sequence features. Therefore, only sex-specific maps were used for these studies.

## Additional files

Additional file 1: Table S1. Summary statistics of 24 linkage groups of sex-specific genetic maps for three families of Asian seabass. (XLSX 21 kb)
Additional file 2: Table S2. Summary statistics of the integrated male and female genetic maps of Asian seabass. (XLSX 11 kb)
Additional file 3: Figure S1. Distribution of F: M ratios for each linkage group throughout three families of Asian seabass. (JPG 318 kb)
Additional file 4: Figure S2. Distribution of the average recombination rates within each category of GC content for male-specific and female-specific maps across families. (JPG 184 kb)

## Abbreviations

GBS: Genotyping-by-sequencing
LG: Linkage group
MAS: Marker-assisted selection
QTL: Quantitative trait locus
