# Supplementary figures and images for: Construction of high-resolution recombination maps in Asian seabass
Source: BMC Genomics. 2017 Jan 10;18:63. doi: 10.1186/s12864-016-3462-z (PMC5223582; doi:10.1186/s12864-016-3462-z)

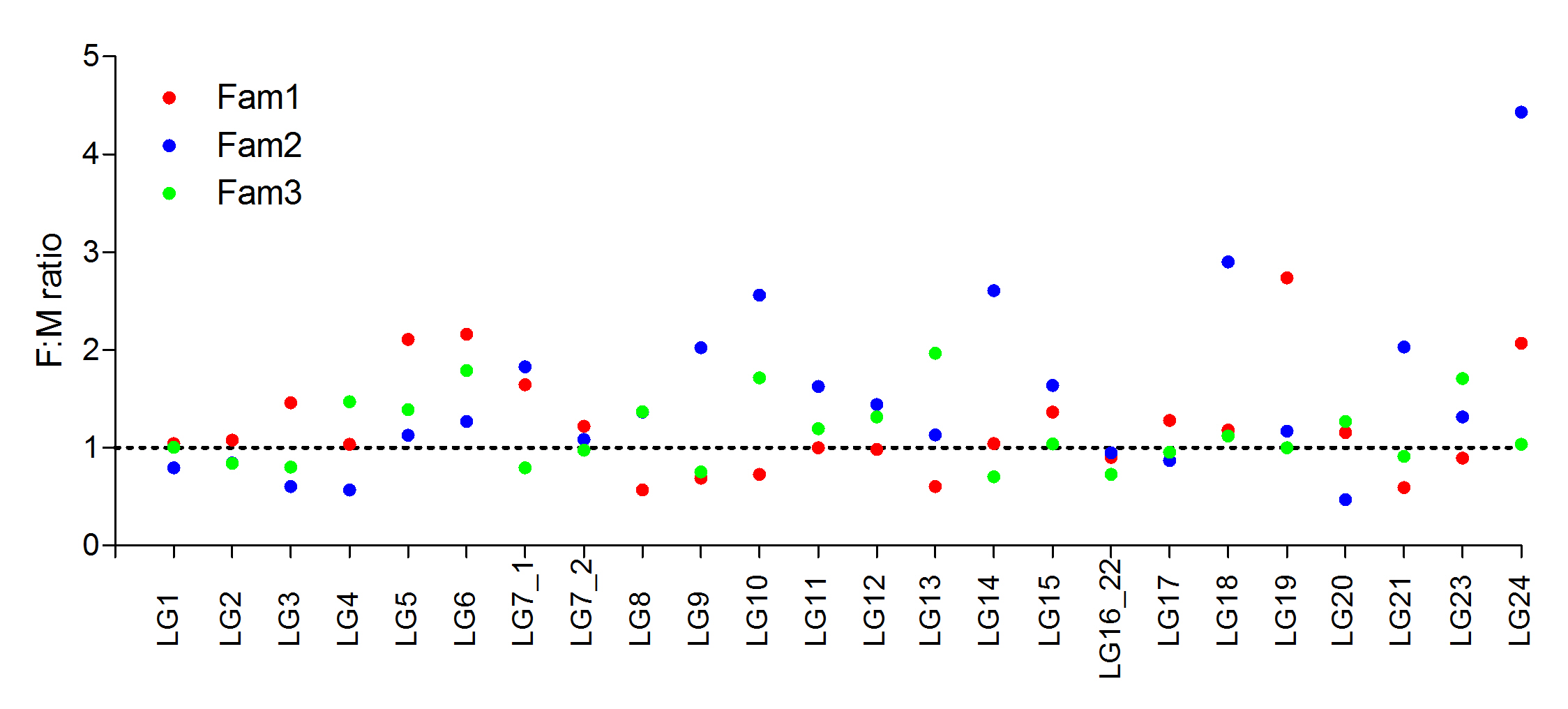

Supplement: Additional file 3: Figure S1. — Distribution of F: M ratios for each linkage group throughout three families of Asian seabass. (JPG 318 kb) [file 12864_2016_3462_MOESM3_ESM.jpg]

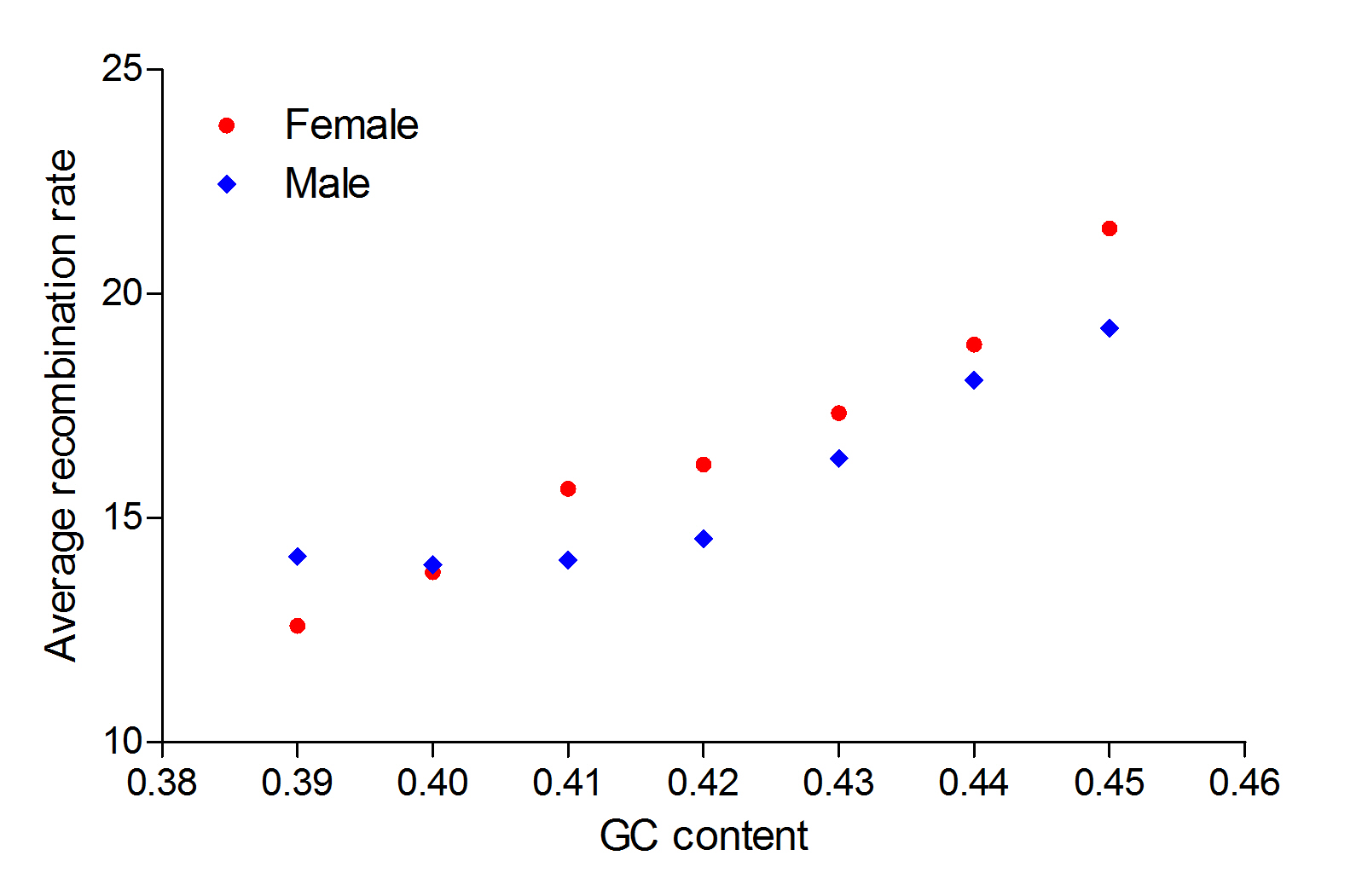

Supplement: Additional file 4: Figure S2. — Distribution of the average recombination rates within each category of GC content for male-specific and female-specific maps across families. (JPG 184 kb) [file 12864_2016_3462_MOESM4_ESM.jpg]
